# Characterization of Hepatocellular Carcinoma Cell Lines Using a Fractionation-Then-Sequencing Approach Reveals Nuclear-Enriched HCC-Associated lncRNAs

**DOI:** 10.3389/fgene.2019.01081

**Published:** 2019-11-08

**Authors:** Eugene Yui-Ching Chow, Jizhou Zhang, Hao Qin, Ting-Fung Chan

**Affiliations:** ^1^School of Life Sciences, The Chinese University of Hong Kong, Shatin, Hong Kong; ^2^State Key Laboratory of Agrobiotechnology, The Chinese University of Hong Kong, Shatin, Hong Kong

**Keywords:** RNA localization, subcellular fractionation, RNA-sequencing, noncoding RNA, hepatocellular carcinoma

## Abstract

**Background:** Advances in sequencing technologies have greatly improved our understanding of long noncoding RNA (lncRNA). These transcripts with lengths of >200 nucleotides may play significant regulatory roles in various biological processes. Importantly, the dysregulation of better characterized lncRNAs has been associated with multiple types of cancers, including hepatocellular carcinoma (HCC). There are many studies on altered lncRNA expression levels, very few, however, have focused on their subcellular localizations, from which accumulating evidences have indicated their close relationships to lncRNA functions. A transcriptome-wide investigation of the subcellular distributions of lncRNAs might thus provide new insights into their roles and functions in cancers.

**Results:** In this study, we subjected eight patient-derived HCC cell lines to subcellular fractionation and independently sequenced RNAs from the nuclear and cytoplasmic compartments. With the integration of tumor and tumor-adjacent RNA-seq datasets of liver hepatocellular carcinoma (LIHC) from The Cancer Genome Atlas (TCGA), *de novo* transcriptome assembly and differential expression analysis were conducted successively and identified 26 nuclear-enriched HCC-associated lncRNAs shared between the HCC samples and the TCGA datasets, including the reported cancer driver *PXN-AS1*. The majority of nuclear-enriched HCC-associated lncRNAs were associated with the survival outcomes of HCC patients, exhibited characteristics similar to those of many experimentally supported HCC prognostic lncRNAs, and were co-expressed with protein-coding genes that have been linked to disease progression in various cancer types.

**Conclusion:** We adopted a fractionation-then-sequencing approach on multiple patient-derived HCC samples and identified nuclear-enriched, HCC-associated lncRNAs that could serve as important targets for HCC diagnosis and therapeutic development. This approach could be widely applicable to other studies into the disease etiologies of lncRNA.

## Introduction

Notorious for a rapid progression, poor prognosis, and limited therapeutic options, hepatocellular carcinoma (HCC) is among the most prevalent types of cancer and causes of cancer-related deaths worldwide. HCC is commonly caused by chronic liver disorders such as viral hepatitis and alcohol-induced cirrhosis (Venook et al., 2010; Maluccio and Covey, 2012). Previous studies suggested that HCC carcinogenesis is a multistep process characterized by genetic alterations and subsequent transcriptome profile dysregulation (Chan et al., 2006; Marquardt et al., 2014). Therefore, elucidation of the underlying mechanisms could potentially improve the diagnosis and management of this disease (Woo et al., 2017).

Significant advancements in our understanding of long non-coding RNA (lncRNA) have been made over the last decade. These non-protein-coding transcripts with lengths of >200 nucleotides are thought to play important regulatory roles in various biological processes *via* diverse mechanisms (Mattick, 2011; Djebali et al., 2012). Recent improvements in RNA deep sequencing technologies and collaborative efforts to augment lncRNA annotation *via* the GENCODE consortium (Harrow et al., 2012) and NONCODE project (Fang et al., 2018) have greatly facilitated the identification and characterization of lncRNA. Importantly, lncRNA has been linked to multiple types of cancer, including HCC (Gibb et al., 2011; Schmitt and Chang, 2016). LncRNAs such as *MALAT1* (Malakar et al., 2017), *HOTAIR* (Gao et al., 2016), *SNHG20* (Zhang et al., 2016), and *HOXD-AS1* (Wang et al., 2017) were reported to be facilitative in HCC tumorigenesis. The dysregulation or ectopic expression of these lncRNAs in tumor cells enhance cancer progression or recurrence and could thus serve as prognostic markers. However, the MiTranscriptome study, which curated thousands of TCGA (The Cancer Genome Atlas) RNA-seq datasets, suggested that the cancer lncRNA transcriptome may be considerably more complex and diverse than the normal transcriptome and that a significant fraction of the lncRNAs expressed by tumor cells might not yet be annotated (Iyer et al., 2015). These findings provide strong motivations for the systematic identification of new HCC-associated lncRNAs through a *de novo* transcriptome assembly of public RNA-seq datasets, as well as additional RNA-seq datasets from in-house samples.

Recently, RNA biologists have expressed interest in the patterns of subcellular lncRNA enrichments. Previous studies have reported that better understood lncRNAs, such as *NEAT1* and *MALAT1* (Sun et al., 2018), are predominantly enriched in nuclear fractions, whereas others such as *H19* and *DANCR* (van Heesch et al., 2014) are enriched in the cytoplasmic fraction. Coincidentally, associations were observed between many lncRNAs with biased subcellular enrichment patterns and HCC. A transcriptome-wide investigation of the subcellular enrichment patterns of HCC-associated lncRNAs might thus provide new insights into the roles and functions of these transcripts in cancers.

In this study, we subjected rRNA-depleted libraries constructed from the cytoplasmic and nuclear fractions of eight patient-derived HCC cell lines to strand-specific RNA sequencing. The cell lines were derived from the HCC tumor tissues of patients of Asian-Chinese ethnicity who had been diagnosed with either chronic hepatitis B/C or nonalcoholic steatohepatitis (NASH). Additionally, 421 HCC patient-derived RNA-seq datasets, including 50 pairs of tumor/tumor-adjacent samples, were downloaded from The Cancer Genome Atlas (TCGA) database (Weinstein et al., 2013). Using both reference-based and *ab initio* approaches to *de novo* transcriptome assembly, we obtained a consensus set of 956 lncRNA annotations across in-house and TCGA samples, of which 450 (47%) had not been described in the GENCODE (v27) annotation. Subsequently, dysregulated lncRNAs were identified through a differential expression analysis and characterized with regard to their subcellular distribution, biological features, and fitness as prognostic markers.

## Materials and Methods

### In House Patient-Derived HCC Cell-Line Samples

HKCI-2, 4, 9, 10, 11, C1, C2, and C3 cell lines were maintained and cultured as described previously. HKCI-2 and 10 cell lines were derived from HCC patients of NASH etiology. HKCI-4,9 and 11 were derived from HCC patients of HBV etiology. HKCI-C1, C2, and C3 were derived from HCC patients of HCV etiology (Gho et al., 2008).

### Subcellular Fractionation and RNA Extraction

The subcellular fractionation methodology was established previously by Djebali et al. in a study of the transcriptional landscape of ENCODE reference human cell lines (Djebali et al., 2012). We used a similar stepwise lysis protocol to generate both cytoplasmic and nuclear fractions from each sample. Briefly, the tissues were initially processed by disrupting the outer cellular membrane to release the cytoplasmic contents. The lysates were subjected to high-speed centrifugation to pellet the intact nuclei (nuclear fraction) from the supernatant (cytoplasmic fraction). A RNeasy MiniElute Cleanup kit (Qiagen) was used to extract the total RNA from the respective fractions. To validate the fractionation protocol, qPCR was conducted to quantitate the relative enrichments of cytoplasmic marker genes (*RPS14*, *GAPDH*) and nuclear marker genes (*MALAT1*) in a control fractionation experiment. qPCR primers were designed to cover exon-exon junctions and to capture only spliced transcripts (**Supplementary Table 1**). Results confirmed >10-fold enrichments of cytoplasmic and nuclear markers in respective fractions, suggesting effective separation of the two subcellular fractions (**Supplementary Figure 1**).

### RNA Sequencing

Total RNA samples were converted into strand-specific, rRNA-depleted libraries, and pair-end sequenced on an Illumina HiSeq 2500 platform by Macrogen Co. (Seoul, South Korea). A total of 7.04 billion reads were generated for eight in-house HCC cell lines. The numbers of clean reads after trimming obtained from cytoplasmic RNA ranged from 59.8 to 99.2 million across eight HCC cell lines and the effective sequencing depth was estimated between 21.3x and 31.6x. The final number of clean reads obtained from nuclear RNA ranged from 95.9 to 171.2 million with the effective sequencing depths between 5.5x and 12.84x. To confirm the outcomes of subcellular fractionation, the expression levels of cytoplasmic marker genes (*RPS14*, *GAPDH*) and nuclear marker genes (*MALAT1, NEAT1, PVT1*) (Chan and Tay, 2018; Sun et al., 2018; Yu et al., 2018) were quantitated from RNA-seq data using featureCounts (version 1.6.3) (Liao et al., 2014). The RNA-seq expression fold changes of *RPS14, GAPDH*, and *MALAT1* were concordant with the qPCR results from the control fractionation experiment. In addition, the RNA-seq expression of *NEAT1* and *PVT1* were enriched in the nuclear fraction by >30-fold and >10-fold respectively. The observation indicated effective separation of the subcellular fractions among all HCC cell line samples (**Supplementary Tables 2A–C**).

### TCGA Datasets

RNA-seq datasets (in FASTQ format) and corresponding clinical data from patients in the TCGA-LIHC cohort were obtained from TCGA following authorization (dbGaP controlled dataset phs000178.v10.p8) (Weinstein et al., 2013). A total of 371 tumor tissue RNA-seq datasets and 50 paired (tumor tissue and tumor-adjacent tissue) RNA-seq datasets were included in subsequent analyses. Among the 50 patients with paired RNA-seq datasets, 7, 5, and 3 of them are with HBV, HCV, and NASH etiology, respectively.

### Transcriptome Assembly

RNA-seq datasets from the eight HCC cell line samples and the 50 paired TCGA-LIHC samples were first trimmed using trimmomatic (Bolger et al., 2014) (version 0.36). For each individual sample:

- The dataset was aligned to the human reference genome (hg38) using STAR (Dobin et al., 2013) (version 2.0.10). After alignment, a reference-based *de novo* transcriptome assembly was conducted using StringTie (Pertea et al., 2015) (version 1.3.3b). The GENCODE v27 annotation was supplied as reference to guide the assembly process.
- The dataset was subjected to *ab initio de novo* transcriptome assembly using Trinity (Grabherr et al., 2011) (version 2.4.0) and GMAP aligner (Wu and Watanabe, 2005).
- A sample-specific transcriptome composed of transcripts assembled by both StringTie and Trinity were constructed using gffcompare (https://github.com/gpertea/gffcompare).

Finally, the transcript merge mode of StringTie was used to unify and merge the sample-specific transcriptomes into a high-consensus transcriptome. The high-consensus transcriptome was composed of transcripts assembled in both HCC cell line samples and TCGA samples.

The assembly support of a transcript was defined as the number of sample-specific transcriptome derived from the 50 paired TCGA samples that included the transcript. The assembly support of transcripts in tumor and tumor-adjacent tissues was counted separately.

### Differential Expression Analysis

The expression level of each transcript was quantified using Kallisto (Bray et al., 2016) (version 0.43.0). EBSeq (Leng et al., 2013), DESeq2 (Love et al., 2014), and edgeR (McCarthy et al., 2012) were used to identify transcripts that were differentially expressed between TCGA LIHC tumor and tumor-adjacent tissue samples. In DESeq2 and edgeR, differentially expressed transcripts were defined as those that satisfied 2 criteria: |log_2_(fold-change)| > 1 and *p* < 0.01 after the Benjamini–Hochberg correction. In EBSeq, differentially expressed transcripts were defined as those with a PPDE (posterior probability that a transcript is differentially expressed) > 0.99 and |log_2_(fold-change)| > 1. Dysregulated transcripts related to HCC progression were defined as the consensus subset of differentially expressed transcripts among the three methods.

### Identification of lncRNA Transcripts

From the merged assembly assemblies, transcripts in non-repeat-masked genomic regions with ≥2 exons, a length >200 nucleotides, available strand information, and an expression level of TPM > 1 were selected. This subset of transcripts was compared with the GENCODE (v27) annotation, and gffcompare was used to assign an annotated or unannotated status. Annotated transcripts classified as “lincRNA” or “antisense” in GENCODE (v27) were considered lncRNAs. Unannotated transcripts were considered lncRNAs if both CPC2 (Kang et al., 2017) and COME (Hu et al., 2017) predicted their coding potential as “noncoding RNA”.

### Survival Analysis

TCGA LIHC patients with clinical survival data were classified into the high-risk or low-risk group based on lncRNA transcript expression. A Kaplan-Meier survival analysis and log-rank test were used to estimate differences in the overall survival times between patients in the 2 groups. All analyses were conducted on the R-3.4.1 framework.

### Promoter Mark Analysis

Processed H3K4me3 ChIP-seq peak calling data from 28 cell lines in 4 categories were obtained from the Human Epigenome Atlas repository (Roadmap Epigenomics Consortium et al., 2015) (Release 9).

- Embryonic stem cells (ESCs): E001, E002, E003, E008, E014, E015, E016, E024
- ESC-derived cells: E004, E005, E006, E007, E009, E010, E011, E012, E013
- Induced pluripotent cells (iPSCs): E018, E019, E020, E021, E022
- ENCODE Cancer cell lines: E114, E115, E117, E118, E123

LncRNA transcripts and genes were considered associated with an active promoter mark if an H3K4me3 ChIP-seq peak was present at ±1000 bp of the 5´ end.

### lncRNA Expression Profiling in Preimplantation Embryonic Cells

Single-cell RNA-seq datasets (in FASTQ format) from human preimplantation embryonic cells in seven stages (oocyte, zygote, 2-cell, 4-cell, 8-cell, morula, and late blastocyst) were downloaded from NCBI SRA (accession: SRP011546) (Yan et al., 2013). Transcript assembly support was calculated as described above. Transcript expression levels were quantified using Kallisto (Bray et al., 2016). LncRNAs were filtered away if no assembly support was detected for the lncRNA transcript and its isoforms in all preimplantation embryonic cell datasets. LncRNAs were considered to be expressed in a preimplantation embryonic stage if a TPM measurement >1 was detected in any cell in ≥2 embryo samples. LncRNAs were further designated as possibly lineage-specific if a TPM >1 was observed in ≥2 cells but <67% of all cells in an embryo sample.

### GO Enrichment Analysis

Differentially expressed, tumor-enriched protein-coding genes with a H3K4me3 active promoter histone mark in ≥4 (of 8) ESC samples were selected and tested for enrichment against a background of all annotated protein-coding genes using GOATOOLS (Klopfenstein et al., 2018). P values were corrected for multiple testing using the Benjamini–Hochberg procedure at an alpha = 0.05.

### Determining the Fractional Enrichment Status of Genes

The FPKM values of each gene in cytoplasmic and nuclear fractions from the eight HCC cell lines were computed using featureCounts (version 1.6.3) (Liao et al., 2014). DESeq2 was used to conduct the in-sample normalization of FPKM values (Love et al., 2014). For each cell line sample, only genes with an expression of ≥0.1 FPKM in both the cytoplasmic and nuclear fractions were retained when computing the log_2_(FPKM_cytoplasmic_ _fraction_/FPKM_nuclear_ _fraction_) metric [abbreviated as log_2_(FPKM_cyto_/FPKM_nuc_)]. A positive log2(FPKM_cyto_/FPKM_nuc_) value indicated a transcript abundance bias towards the cytoplasmic fraction, while a negative value indicated a bias towards the nuclear fraction.

The list of human housekeeping genes was obtained from a previous study (Eisenberg and Levanon, 2013). The 5^th^ and 95^th^ percentiles of the mean log_2_(FPKM_cyto_/FPKM_nuc_) values of these housekeeping genes were selected as the lower and upper reference limits, respectively, for non-fraction-specific genes. Genes with log_2_(FPKM_cyto_/FPKM_nuc_) measurements below the lower limit in ≥4 cell lines were considered nuclear-enriched, while those with measurements exceeding the upper limit in ≥4 cell lines were considered cytoplasmic-enriched.

The mean log_2_(FPKM_cyto_/FPKM_nuc_) values for the cytoplasmic marker genes *GAPDH* and *RPS14* were 3.860 and 3.306, and mean value for the nuclear marker gene *MALAT1* was -4.132. Our proposed metric cutoff scheme suggested cytoplasmic-enrichment status of *GAPDH* and *RPS14* and the nuclear-enrichment status of *MALAT1*. The findings from fractionation-then-sequencing data agreed with the results of prior qPCR experiments.

### Co-expression Analysis

The co-expression analysis was performed using the Weighted Correlation Network Analysis (WGCNA) R package (Langfelder and Horvath, 2008) and was based on expression data from all protein-coding and lncRNA genes (including unannotated lncRNAs assembled in this study) quantified from 421 tumor datasets in the TCGA-LIHC cohort using Kallisto (Bray et al., 2016). Genes expressed in <20% of datasets were excluded from the analysis. Subsequently, pairwise Pearson’s correlation coefficients (PCCs) were calculated between 26 nuclear-enriched HCC-associated lncRNA genes and all other genes. Genes exhibiting significant co-expression with nuclear-enriched HCC-associated lncRNA genes (PPC ≥ 0.6, *p* < 0.05) were selected for a hierarchical clustering analysis using Python.

## Results

### *De Novo* Transcriptome Assembly Generates Consensus HCC lncRNA Catalogue

**Figure 1:**
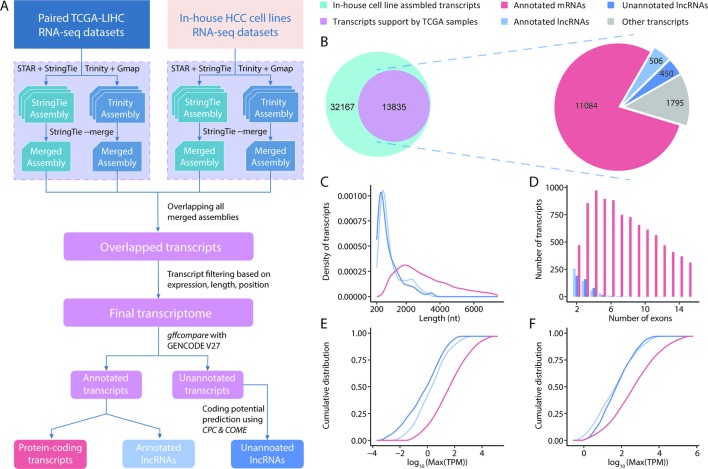
Overview of the transcriptome assembly and lncRNA annotation process. **(A)** Computational pipeline for the identification of high-consensus lncRNAs from TCGA-LIHC and in-house samples. **(B)** Venn diagram showing the overlap of transcripts assembled from in-house and TCGA-LIHC samples. Pie graph of the distributions of overlapped transcripts according to category. Comparisons of the transcript lengths, exon numbers and expression of newly assembled lncRNAs, GENCODE-annotated lncRNAs, and protein-coding transcripts: **(C)** Transcript length distribution; **(D)** Bar graph of the exon numbers of transcripts in different groups; **(E**,**F)** Cumulative distribution curve of the maximum expression of each transcript in **(E)** tumor and **(F)** tumor-adjacent tissues.

Initially, we conducted a transcriptome assembly to detect lncRNAs in HCC samples (**Figure 1A**). The resolution of RNA-seq data from TCGA is not sufficient to decode clear strands for assembled transcripts because of a lack of strand information. We overcame this limitation by integrating a set of strand-specific RNA-seq data from in-house HCC cell lines into our pipeline. A total of 216 unique transcriptome assemblies were produced from 108 datasets (8 from in-house HCC cell lines, 100 from 50 pairs of TCGA-LIHC tumor/tumor-adjacent samples). The reference-based and *ab initio* transcriptome assemblies were incorporated using StringTie (Pertea et al., 2015) and Trinity (Grabherr et al., 2011), respectively. To generate a high-consensus transcriptome, 13, 835 multi-exonic transcripts (including 10, 591 genes) with definitive strand information identifiable by both StringTie and Trinity in both types of datasets were extracted for downstream analyses. These datasets included 506 lncRNA transcripts (444 genes) and 11,084 protein-coding transcripts (9, 038 genes) annotated by GENCODE (v27). Using the reference lncRNA annotation from GENCODE, we identified 450 high-quality unannotated lncRNA transcripts (411 genes) with support from CPC2 (Kang et al., 2017) and COME (Hu et al., 2017) (**Figure 1B**). These novel lncRNA transcripts were first divided into 4 categories based on the “transfrag class codes” defined by gffcompare (**Supplementary Table 3**). Specifically, > 50% of lncRNAs were assigned a class code “j” and were identified as potential isoforms that shared at least 1 splice junction with reference transcripts. Furthermore, 24.4% and 10.2% of lncRNAs were assigned class codes “u” and “i” and were classified as intergenic and intronic transcripts, respectively. The remaining 15% of lncRNAs were assigned class codes of “x”, “o”, and “c,” indicating lncRNAs that overlapped with currently annotated transcripts in either strand. Through a comparison with other noncoding annotations, we observed that approximately half of our newly assembled lncRNAs were not previously reported. For example, the MiTranscriptome (Iyer et al., 2015) and NONCODE (v5) (Fang et al., 2018) annotations yielded overlap rates of 55.5% and 41%, respectively, with the novel lncRNAs in our study (**Supplementary Figure 2**). Accordingly, our RNA-seq data seemed to have a sufficient sequencing depth for capturing novel transcripts.

Using the assembled transcripts from our annotation, we revisited the basic characteristics of lncRNAs and protein-coding transcripts. Previous studies mentioned that lncRNAs have a shorter length, lower expression, and smaller exon number than protein-coding transcripts. To address whether the newly assembled lncRNAs also exhibited these features, we compared 450 unannotated lncRNAs with the protein-coding transcripts and annotated lncRNAs in our final transcriptome annotation. We observed that our newly assembled lncRNA transcripts were significantly shorter in length than protein-coding transcripts (*p* < 0.001, Student’s t-test), but were comparable to the lncRNAs annotated in GENCODE (**Figure 1C**). We observed similar results in comparisons of exon numbers and expression (**Figures 1D–F**). Taken together, these results suggest that the sequencing data and lncRNA identification scheme applied in this study allowed us to assemble highly credible lncRNAs with considerable lengths and expression levels.

### Differential Expression Analysis Reveals Tumor-Specific lncRNA Upregulation

**Figure 2:**
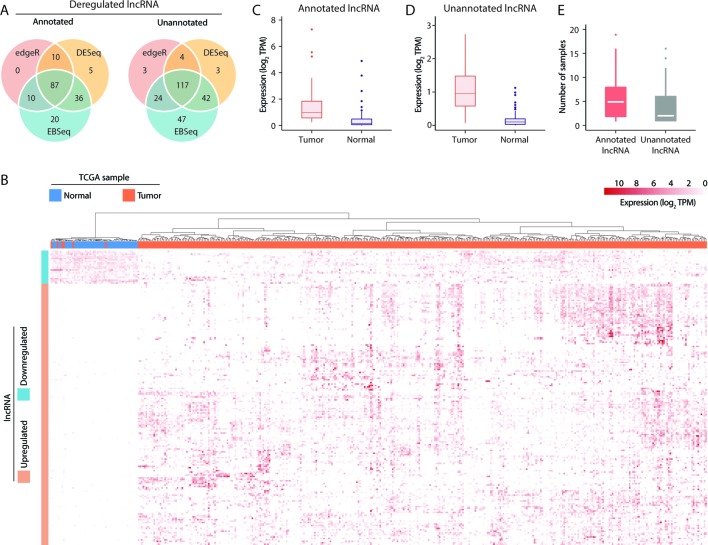
Visualization of long noncoding RNA (lncRNA) dysregulation in hepatocellular carcinoma (HCC). **(A)** Dysregulated annotated and unannotated lncRNAs identified using 3 methods of differential expression analysis. **(B)** Hierarchical clustering of TCGA-LIHC samples based on the expression of dysregulated lncRNAs. **(C)** Median transcript expression of annotated differentially expressed tumor-specific lncRNAs in tumor and tumor-adjacent TCGA-LIHC samples. **(D)** Median transcript expression of unannotated differentially expressed tumor-specific lncRNAs in tumor and tumor-adjacent TCGA-LIHC samples. **(E)** Transcript assembly support in the subset of tumor-specific lncRNAs assembled exclusively in tumor samples.

To identify lncRNAs with broad involvement in the prognosis of HCC, we conducted differential expression tests of tumor and tumor-adjacent samples in TCGA using the EBSeq (Leng et al., 2013), DESeq2 (Love et al., 2014) and edgeR (McCarthy et al., 2012) methods. Notably, these methods yielded comparable results. Overall, 87 and 117 deregulated lncRNAs predicted by all 3 methods were identified in the annotated and unannotated lncRNA sets, respectively (**Figure 2A**). To validate the outcomes of a differential expression analysis, all 421 RNA-seq datasets from the 371 patients in the TCGA-LIHC cohort were clustered according to the expression profiles of all dysregulated lncRNAs. We observed that the upregulated and downregulated lncRNAs could be used effectively to separate tumor and nontumor samples (*p* < 0.001, chi-square test) (**Figure 2B**), indicating that the differentially expressed lncRNA set detected in this study was reliable.

Of the 204 deregulated lncRNAs, 105 unannotated and 76 annotated transcripts were upregulated in tumor samples. Nevertheless, low but detectable levels of expression in the tumor-adjacent samples (**Figures 2C, D**) suggest that the biological functions of some lncRNAs might not be specific to cancer-related mechanisms. Alternatively, as TCGA datasets are non-strand-specific, this observation might also be attributable to artifacts from transcript quantification. To evaluate the tumor specificity of lncRNA accurately, we revisited our transcriptome assembly and calculated the assembly support for each within the 50 pairs of TCGA-LIHC samples. As a successful assembly requires moderate and uniform sequence coverage across the entire transcript region, assembly support may be a better and more stringent indicator of the presence of transcripts. Here, 81/45 annotated/unannotated and tumor-upregulated lncRNAs were found to have no assembly support from tumor-adjacent samples. These lncRNAs were supported by up to 19 TCGA-LIHC tumor samples (**Figure 2E**), coherent with the general observation that lncRNA expression is highly heterogeneous across tumor samples. Results of differential expression analysis and the assembly support of these lncRNAs were listed in **Supplementary Table 4**.

### Subcellular Transcript Distributions Reveal Nuclear-Enriched HCC-Associated lncRNAs

Next, the gene expression landscapes in the nuclear and cytoplasmic fractions were investigated using fractionation-then-sequencing data from in-house HCC cell lines. To evaluate the relative transcript abundances between the two fractions, the normalized gene expression (in FPKM) in the cytoplasmic fraction were divided by the expression in the nuclear fraction to obtain a log_2_(FPKM_cyto_/FPKM_nuc_) value for each gene. A positive log_2_(FPKM_cyto_/FPKM_nuc_) value indicated a transcript abundance bias toward the cytoplasmic fraction, while a negative value indicated a bias towards the nuclear fraction.

**Figure 3:**
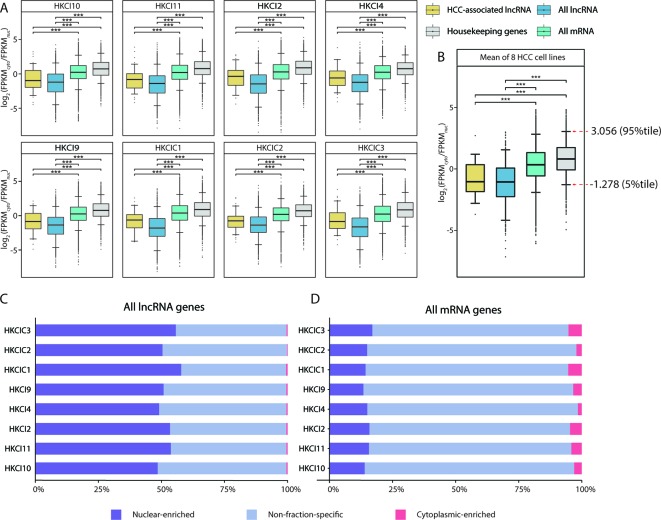
Subcellular distribution of RNAs in eight in-house hepatocellular carcinoma (HCC) cell lines. **(A)** Comparison of the RNA fraction distributions (gene expression FPKM ≥ 0.1 in both cytoplasm and nucleus) in different categories from eight in-house HCC cell lines. **(B)** Box plot of the RNA subcellular distribution based on the mean expression across eight cell lines. Pairwise comparisons were conducted using Mann-Whitney U test. All long noncoding RNA (lncRNA): All lncRNA genes (antisense and lincRNA biotype) annotated in GENCODE (v27); All mRNA: All mRNA genes annotated in GENCODE (v27); HCC-associated lncRNA: All HCC-associated lncRNAs revealed in this study; Housekeeping genes: Genes expressed uniformly across diverse tissues according to a study by *Eisenberg et al.* **(C**, **D)** Distributions of mRNA and lncRNA genes according to enrichment patterns (nuclear-enriched/cytoplasm-enriched/non-fraction-specific) in subcellular fractions.

The overall mRNA population was not apparently biased toward either fraction, with a median log_2_(FPKM_cyto_/FPKM_nuc_) close to 0. In contrast, the overall lncRNA population was significantly more biased toward the nuclear fraction (*p* < 0.001, Mann-Whitney U test), with a negative median log_2_(FPKM_cyto_/FPKM_nuc_). Moreover, the HCC-associated lncRNAs identified in this study also resembled the characteristics of nuclear bias from the overall lncRNA population (**Figure 3A**). These observations were also consistent when the measurements of the eight HCC cell lines were averaged (**Figure 3B**) and were consistent with findings from previous fractionation-then-sequencing studies of other cell lines, such as HepG2 (Benoit Bouvrette et al., 2018).

Nevertheless, fraction bias in individual transcripts might be due to temporal fluctuations in cellular transcript levels (Blake et al., 2003; Kaern et al., 2005; Raj and van Oudenaarden, 2008; Eldar and Elowitz, 2010) or due to mechanisms such as burst transcription (Dar et al., 2012; Bahar Halpern et al., 2015a; Bahar Halpern et al., 2015b), rather than genuine fraction-enrichment events. To select the most confident fraction-enriched subset of genes, a list of 3,804 RNA-seq-derived human housekeeping genes was downloaded (Eisenberg and Levanon, 2013). Of these, 3,543 (93.1%) are mRNA genes and consistently expressed (FPKM ≥ 0.1) in both the nuclear and cytoplasmic fractions of all eight HCC cell lines. The mean log_2_(FPKM_cyto_/FPKM_nuc_) values of these housekeeping genes were then assumed a reference range for normal variations in fraction bias. This implied that only genes with a substantially negative or positive log_2_(FPKM_cyto_/FPKM_nuc_) values would be considered fraction-enriched. Based on this assumption, the 5^th^ and 95^th^ percentiles of the reference range (-1.278–3.056) were selected as the cutoffs for detecting nuclear-enriched and cytoplasmic-enriched genes. Asymmetry in the cutoff values was consistent with the general knowledge that mRNAs are predominantly enriched in the cytoplasmic fraction, which is also the site of protein translation (**Figure 3B**).

**Figure 4:**
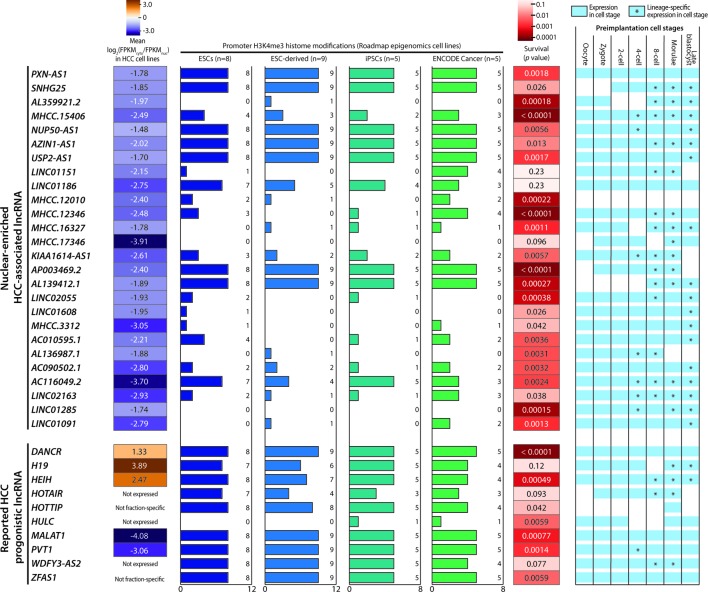
Characteristics of nuclear-enriched hepatocellular carcinoma (HCC)-associated long noncoding RNAs (lncRNAs). Summary of the subcellular fraction enrichment status, associated H3K4me3 histone modifications, survival analysis and expression profiles in preimplantation embryonic cells of 26 nuclear-enriched HCC-associated lncRNA genes, and 10 reported HCC prognostic lncRNA genes. Genes with a FPKM < 0.5 in ≥5 cell lines were marked as “Not expressed”.

Based on our cutoff scheme, approximately 80% of the overall mRNA population was considered non-fraction-specific (**Figure 3C**). In contrast, >50% of the lncRNA population appeared to be fraction-enriched, with a strong bias toward enrichment in the nuclear fraction (**Figure 3D**). Of the 126 proposed HCC-associated lncRNA transcripts, 28 transcripts corresponding to 26 lncRNA genes (**Supplementary Table 5**) exhibited stable nuclear-enrichment in ≥4 in-house HCC cell lines (**Figure 4A**). Remarkably, the list of genes included *PXN-AS1*, which was reported to interact with the nuclear-localized splicing factor *MBNL3* to promote liver and lung cancer progression (Yuan et al., 2017). Although six of the proposed nuclear-enriched HCC-associated lncRNA genes were not annotated in the GENCODE reference, all had corresponding entries in the MiTranscriptome catalog, and *MHCC.16327* and *MHCC.17346* were also suggested to be dysregulated in liver cancer by MiTranscriptome study (Iyer et al., 2015) (**Supplementary Table 6**).

### Characterization of Nuclear-Enriched HCC-Associated lncRNAs Supports Potential Cancer-Driving Capabilities

The 26 nuclear-enriched HCC-associated lncRNAs were further characterized in a nonexclusive manner for histone marks on active promoters and correlations with patient survival and embryonic tissue expression. First, we investigated H3K4me3 histone marks on the candidate lncRNAs. H3K4me3 indicates the presence of active promoter structures (Mikkelsen et al., 2007) and is used widely to support lncRNA expression in cell or tissue samples (Guttman et al., 2009; Marques et al., 2013). We focused on H3K4me3 marks associated with stem cells, as some cancer cells may express lncRNAs ectopically to acquire a stem-like phenotype (Jiang et al., 2016). Strikingly, all GENCODE-annotated candidates possessed stem cell-associated H3K4me3 marks and seven, including *PXN-AS1*, were found to possess H3K4me3 marks in all surveyed ESCs, ESC-derived cells, and iPSCs (**Figure 4**).

To test our hypothesis, we downloaded and evaluated a set of experimentally supported HCC prognostic lncRNA from the Lnc2Cancer database (Ning et al., 2016). Of the 10 reported HCC-prognostic lncRNAs, the majority possessed stem-cell associated H3K4me3 (**Figure 4**). Moreover, a gene ontology (GO) analysis of 3, 295 tumor-enriched protein-coding genes with stem-cell associated H3K4me3 marks also revealed the significant enrichment of GO terms related to stem cell functions such as cell cycle, cell division, and cell adhesion (**Supplementary Figure 3**).

Next, the candidate lncRNA expression patterns in human preimplantation embryonic cells were surveyed based on published single-cell RNA-seq datasets (Yan et al., 2013). This analysis was motivated by the observation that the majority of the 10 reported HCC prognostic lncRNAs were expressed and regulated along the course of preimplantation development (**Figure 4**). This pattern suggests the possible superimposition of lncRNA functions on developmental regulation and cancer progression (Janic et al., 2010; Planells-Palop et al., 2017; Bruggeman et al., 2018). Excitingly, all nuclear-enriched HCC-associated lncRNAs were expressed in some preimplantation stages, consistent with reports of prognostic lncRNA. Moreover, these transcripts exhibited possible lineage specificity because they were expressed in only a fraction of embryonic cells at different stage(s), typically beginning at the 4-cell or 8-cell stage (**Figure 4**).

Lastly, we conducted a survival analysis based on the clinical data of TCGA-LIHC patients. Twenty-three of 26 candidates significantly affected the survival outcomes of TCGA HCC patients (*p* < 0.05) and could serve as potential prognostic markers (**Figure 4**). Overall, this characterization analysis suggested that the 26 nuclear-enriched HCC-associated lncRNAs identified in our bioinformatics pipeline shared the characteristics of experimentally supported HCC-prognostic lncRNAs and may also possess cancer-driving capabilities.

### Co-expression Analysis Connects HCC-Associated lncRNAs With Cancer-Driving Protein-Coding Genes

**Figure 5:**
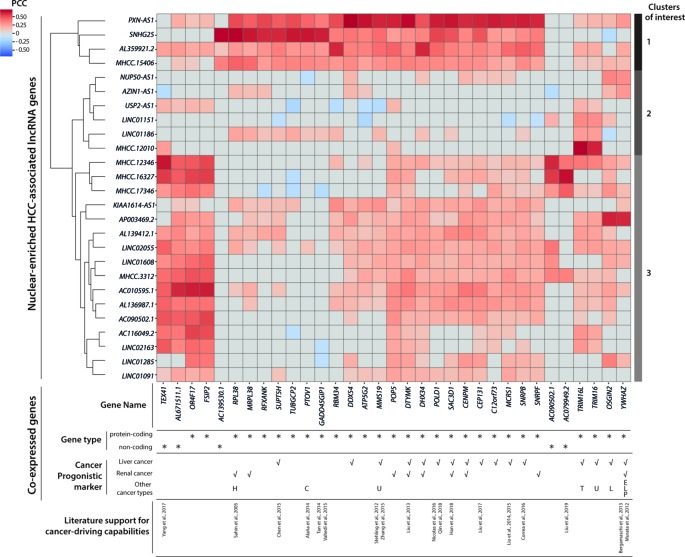
Co-expression and clustering analysis of nuclear-enriched hepatocellular carcinoma (HCC)-associated long noncoding RNAs (lncRNAs). Heatmap of pairwise co-expression correlations (in Pearson’s correlation coefficients; PCC) between 26 nuclear-enriched HCC-associated lncRNAs and 33 co-expressed genes. Cancer prognostic marker information was downloaded from the Human Protein Atlas database. Abbreviations for other cancer types; C, Colorectal cancer; E, Endometrial cancer; H, Head and neck cancer; L, Lung cancer; P, Pancreatic cancer; T, Thyroid cancer; U, Urothelial cancer.

To connect HCC-associated lncRNAs with their potential biological functions, we conducted a co-expression analysis and identified 33 partner genes that were strongly co-expressed (PCC ≥ 0.6, *p* < 0.05) with some of the 26 nuclear-enriched HCC-associated lncRNA genes (**Figure 5**). Of the 33 co-expression partner genes, more than half were suggested cancer prognostic markers (Uhlen et al., 2017), while 13 were identified as possible cancer drivers by either experimental studies or non-TCGA based bioinformatics studies. Subsequently, hierarchical clustering was conducted to group HCC-associated lncRNAs into three clusters based on their co-expression partners (**Figure 5**):

*Cluster 1*: This included *PXN-AS1* and three other lncRNAs of which gene expression was associated with multiple cancer-driving, protein-coding genes. The findings suggest that these genes may be upstream regulators of HCC and would thus influence the expression of downstream cancer-driving genes. These transcripts would be the most promising novel HCC-driving lncRNA candidates.
*Cluster 2*: Except for *MHCC.12010*, which was strongly co-expressed with *TRIM16* and *TRIM16L*, the genes in cluster 2 were not significantly co-expressed with other genes. Nevertheless, past experimental studies have supported *TRIM16* as a *de facto* tumor suppressor (Marshall et al., 2010) that could inhibit cell migration and invasion in HCC (Li et al., 2016). Overall, the potential functions of cluster 2 lncRNAs in HCC remained unclear because of the absence of co-expression partners. These were the novel HCC-driving lncRNA candidates with the least confidence.
*Cluster 3*: Despite substantially weaker PCC values, the majority of lncRNAs in cluster 3 were co-expressed with cancer-driving genes that had also been correlated with the gene expression of *PXN-AS1*/cluster 1 lncRNAs. These findings suggested that cluster 3 also included viable novel HCC-driving lncRNA candidates. Among these candidates, *MHCC.16327* and *AP003469.2* were strongly co-expressed with *AC079949.2* and *YWHAZ*, respectively, and both genes were reported to be cancer-drivers (Murata et al., 2012; Bergamaschi et al., 2013; Liu et al., 2019).

Overall, our co-expression analysis established connections between some nuclear-enriched, HCC-associated lncRNAs, and reported cancer-driving protein-coding genes. Moreover, our findings enabled the prioritization of more promising lncRNA candidates for further experimental evaluations. The co-expression partners of these candidates provided clues regarding the cellular mechanisms that might involve the candidate lncRNAs.

## Discussion

The use of patient-derived HCC cell lines is a major highlight of this study and distinguishes it from the common approach based on surgically removed tumor tissues. As cancer cell line cultures are presumably more homogeneous (Gillet et al., 2013; Goodspeed et al., 2016) than cancer tissue samples (Fidler and Hart, 1982; Michor and Polyak, 2010; Friedl and Alexander, 2011), the assembled transcriptome would more likely provide a genuine reflection of HCC biology and lack contamination from transcripts expressed exclusively in normal tissues. Meanwhile, the inclusion of a TCGA dataset not only compensated our inability to perform a differential expression analysis of transcripts upregulated selectively in tumor cells, but also helped to elucidate a subset of HCC-associated transcripts that were commonly expressed in patients of different ethnicities and clinical backgrounds. While associations between the transcripts and any particular etiology of HCC should not be assumed based on results presented in this study, a substantial variation of expression values of these transcripts were observed among samples of different etiologies (**Supplementary Tables 7**, **8**), hinting that it may be desirable to conduct further investigations into the candidate HCC-associated transcripts in an etiology-aware manner.

This study applied a novel approach in which the de novo transcriptome assembly technique was applied to validate the presence of lncRNAs in samples. Accordingly, the analysis extended beyond the usual scope of identifying novel transcripts. This approach may trade sensitivity for specificity, however, as the successful rediscovery of transcripts with *de novo* assemblies requires moderate and even sequencing read coverage over the entire transcript length and most splice junctions. Moreover, both misassembled transcripts specific to either the *ab initio* assembly (Trinity) or reference-based assembly (StringTie) approaches could be effectively removed by intersecting the 2 assemblies. Together, these procedures reduce the chance of detecting lncRNA from artifacts in bioinformatics processes.

The subcellular localization of transcripts, especially noncoding RNAs, has increasingly attracted attention from RNA biologists. Notable studies of examples such as *MALAT1* and *NEAT1* (Sun et al., 2018) suggested that RNA post-transcriptional regulation could occur extensively within the cell nucleus. Importantly, these observations coincided with the strong nuclear-enrichment of these transcripts, suggesting that the systematic determination of subcellular transcript enrichment using a fractionation-then-sequencing approach could help to identify regulatory (non-coding) transcripts for which the site-of-action resided specifically within the nucleus or cytoplasm. In this study, we demonstrated that while most transcripts exhibit varying degrees of uneven distribution between the cytoplasmic and nuclear fractions, only a minority of transcripts (mRNAs and lncRNAs) exhibit drastic fractional enrichment comparable to the hallmark examples.

In contrast to previous fractionation-then-sequencing studies that evaluated only individual cell lines, our study newly reveals that the subcellular distribution biases of transcripts are relatively stable, at least within a population of HCC cell lines with varied genetic and clinical backgrounds. The findings suggest that the tight regulation of subcellular transcript distributions might be crucial for the maintenance of cellular homeostasis in both healthy and cancerous cells.

Additionally, our fractionation sequencing results also indicates that a small but significant proportion of mRNA genes could be nuclear-enriched. Given that protein-coding capability are *a posteriori* property of mRNAs defined by translational machinery, it might be possible for mRNA to attain lncRNA-like regulatory roles during their transient stay or intentional retainment within the cell nucleus (Carmody and Wente, 2009; Bahar Halpern et al., 2015a), where they are unlikely to be translated. Furthermore, the presence of complex mechanisms to control nuclear RNA levels (Schmid and Jensen, 2018) also hints potential implications for the subset of transcripts that are selectively retained in the compartment. Deeper explorations into RNA subcellular localization landscape may offer insights to the biological significance of nuclear-enriched transcripts and hold promises to further elucidate nuclear RNA biology.

## Data Availability Statement

The raw sequencing data and clinical survival data for 371 HCC patients in the TCGA-LIHC cohort are available at GDC data portal (https://portal.gdc.cancer.gov/ ) upon request from dbGaP under accession phs000178.v10.p8. Single-cell RNA-seq data from human preimplantation embryonic cells was obtained from NCBI SRA database under the accession SRP011546. Processed H3K4me3 ChIP-seq peak calling data was retrieved from the Human Epigenome Atlas repository (Roadmap Epigenomics Consortium et al., 2015) (release 9). The subcellular fractionation RNA-seq data for eight HCC cell lines is available at NCBI SRA database under the accession PRJNA543441.

## Author Contributions

T-FC managed the project. T-FC and HQ designed the experiments. HQ conducted the experiments. T-FC, EC, and JZ designed the bioinformatics analysis. EC and JZ conducted the bioinformatics analysis. T-FC, EC, and JZ wrote the manuscript.

## Funding

This work is partially supported by the CUHK Direct Grants 4053242 and 4053364, a General Research Fund (14102014) from the Research Grants Council to T-FC, and a funding from the Innovation and Technology Commission, Hong Kong Government to the State Key Laboratory. EC is supported by the Hong Kong PhD Fellowship Scheme.

## Conflict of Interest

The authors declare that the research was conducted in the absence of any commercial or financial relationships that could be construed as a potential conflict of interest.
