# Supplementary material for: Characterization of Hepatocellular Carcinoma Cell Lines Using a Fractionation-Then-Sequencing Approach Reveals Nuclear-Enriched HCC-Associated lncRNAs
Source: Front Genet. 2019 Nov 8;10:1081. doi: 10.3389/fgene.2019.01081 (PMC6857473; doi:10.3389/fgene.2019.01081)
Supplement: Supplementary file 1 [file DataSheet_1.docx]

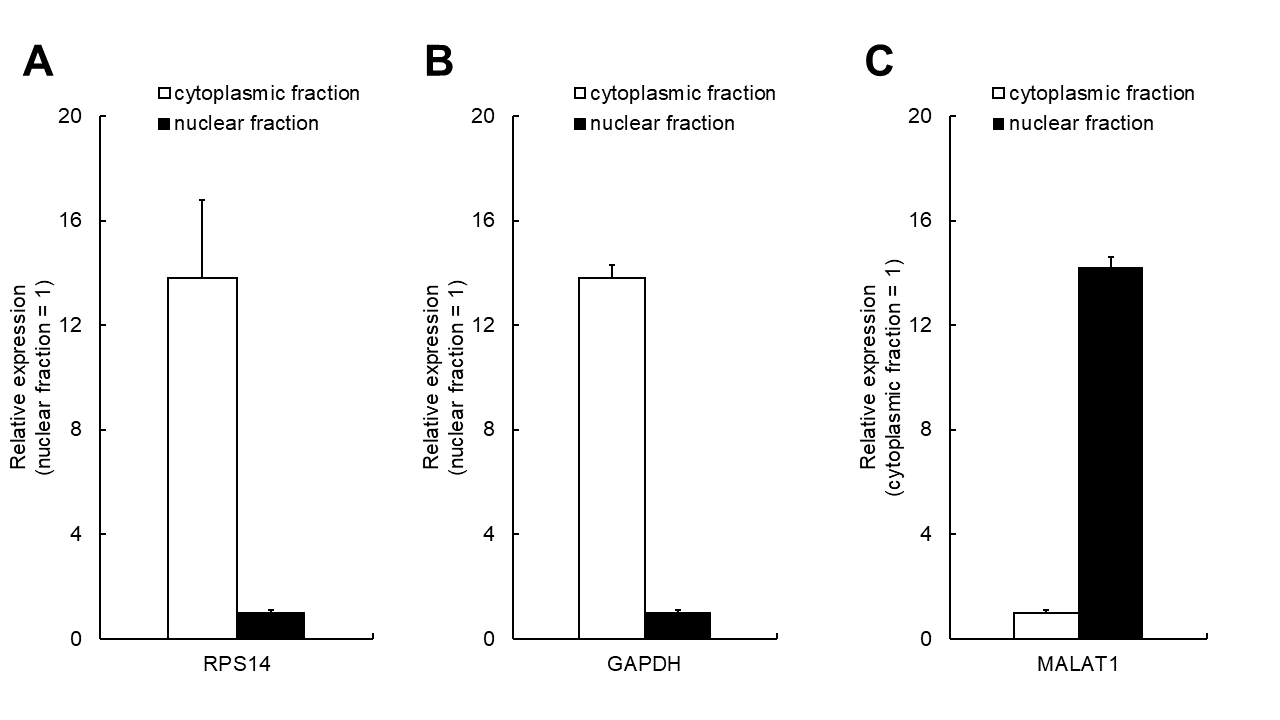


**Supplementary Figure 1:** A control sub-cellular fractionation experiment was carried out to validate the fractionation protocol. Gene expressions of (a) *RPS14,* (b) *GAPDH* and (c) *MALAT1* was measured by qRT-PCR. *RPS14* and *GAPDH* were used as cytoplasmic markers and *MALAT1* was used as a nuclear marker. Results indicated >10-fold enrichments of the marker genes in respective fractions, suggesting effective separation of the two subcellular fractions.


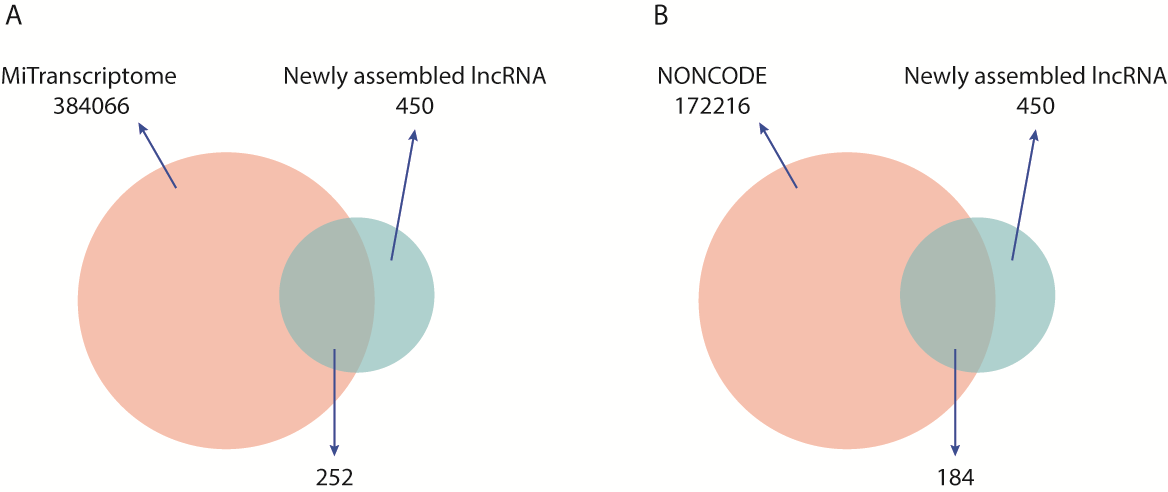


**Supplementary Figure 2.** Venn diagram showing the overlap between our newly assembled lncRNA and other lncRNA databases: (A) MiTranscriptome, (B) NONCODE V5.


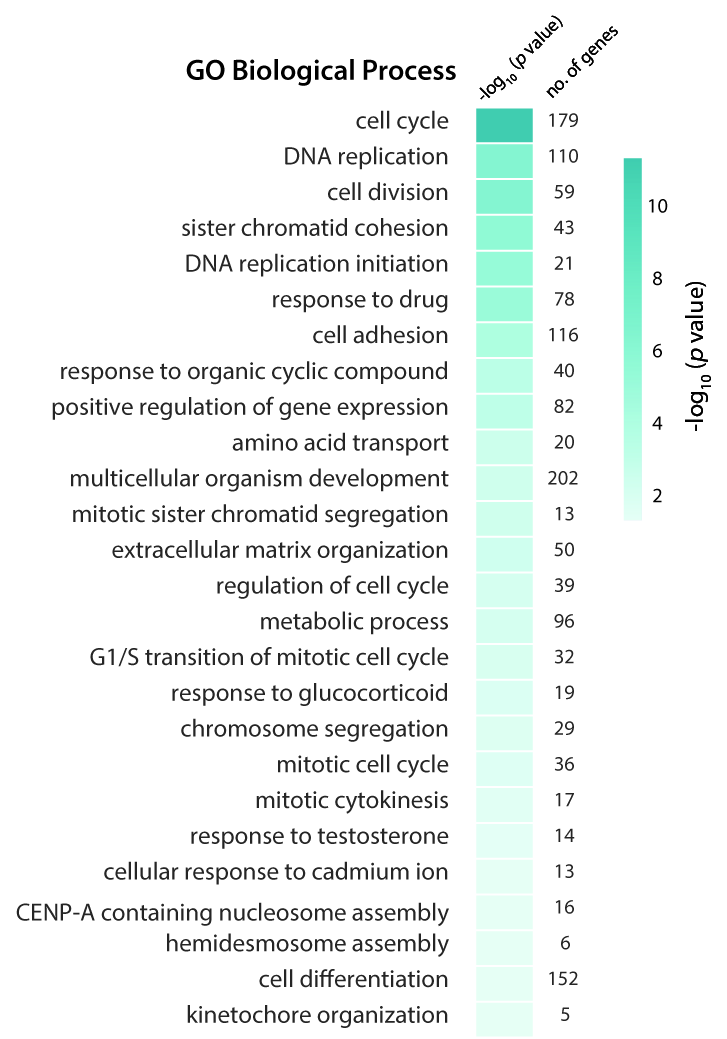


**Supplementary Figure 3.** GO enrichment analysis of 3,295 tumor-enriched protein coding genes with stem-cell associated H3K4me3 marks

**Supplementary Table 1:** qPCR primer sequences

| **Gene** | **Forward primer** | **Reverse primer** | **References** |
| --- | --- | --- | --- |
| ***RPS14*** | 5' GGCAGACCGAGATGAATCCTC 3' | 5' CAGGTCCAGGGGTCTTGGTCC 3' | (Hauber et al., 1995) |
| ***GAPDH*** | 5’ TGCACCACCAACTGCTTAGC 3’ | 5’ GGCATGGACTGTGGTCATGAG 3’ | (Vandesomlele, 2002) |
| ***MALAT1*** | 5' TGGTAGCTTTTGTATTATCAAACTTT 3' | 5' CTGCCAGGCTGGTTATGACT 3' | (Meseure et al., 2016) |

**Supplementary Table 2A:** Gene expression levels (in FPKM) of subcellular fraction marker genes in RNA-seq data

| **Cell line** | **HKCI10** | | **HKCI11** | | **HKCI2** | | **HKCI4** | | **HKCI9** | | **HKCIC1** | | **HKCIC2** | | **HKCIC3** | |
| --- | --- | --- | --- | --- | --- | --- | --- | --- | --- | --- | --- | --- | --- | --- | --- | --- |
| **Subcellular Fraction** | **Cyto- plasmic** | **Nuclear** | **Cyto- plasmic** | **Nuclear** | **Cyto- plasmic** | **Nuclear** | **Cyto- plasmic** | **Nuclear** | **Cyto- plasmic** | **Nuclear** | **Cyto- plasmic** | **Nuclear** | **Cyto- plasmic** | **Nuclear** | **Cyto- plasmic** | **Nuclear** |
| **GAPDH** | 451.73 | 41.72 | 1,658.38 | 91.40 | 2,087.50 | 108.72 | 1,104.34 | 139.57 | 933.21 | 64.05 | 5,419.62 | 243.87 | 1,010.54 | 89.93 | 1,676.71 | 92.10 |
| **RPS14** | 78.26 | 10.44 | 77.82 | 7.83 | 131.67 | 10.14 | 86.11 | 12.96 | 56.35 | 6.60 | 77.64 | 5.27 | 41.52 | 4.58 | 109.20 | 8.71 |
| **MALAT1** | 65.04 | 613.13 | 60.56 | 840.69 | 63.78 | 2,296.46 | 73.48 | 1,418.21 | 68.68 | 1,709.48 | 121.64 | 2,181.67 | 63.33 | 868.97 | 69.59 | 1,116.08 |
| **NEAT1** | 3.05 | 106.15 | 4.06 | 604.45 | 2.91 | 428.14 | 3.05 | 164.95 | 4.78 | 515.53 | 16.30 | 3,418.56 | 5.17 | 315.24 | 4.56 | 326.88 |
| **PVT1** | 0.84 | 11.01 | 2.28 | 28.14 | 1.75 | 24.28 | 1.00 | 15.15 | 0.58 | 7.61 | 0.40 | 7.11 | 1.32 | 14.05 | 0.97 | 11.80 |

**Supplementary Table 2B:** Gene expression fold changes of cytoplasmic fraction marker genes in RNA-seq data

| **Cell line / Expression fold change  (Cytoplasmic compared to Nuclear)** | **HKCI10** | **HKCI11** | **HKCI2** | **HKCI4** | **HKCI9** | **HKCIC1** | **HKCIC2** | **HKCIC3** |
| --- | --- | --- | --- | --- | --- | --- | --- | --- |
| **RPS14** | 10.83 | 18.14 | 19.20 | 7.91 | 14.57 | 22.22 | 11.24 | 18.21 |
| **GAPDH** | 7.50 | 9.94 | 12.99 | 6.64 | 8.54 | 14.73 | 9.07 | 12.54 |

**Supplementary Table 2C:** Gene expression fold changes of nuclear fraction marker genes in RNA-seq data

| **Cell line / Expression Fold change  (Nuclear compared to Cytoplasmic)** | **HKCI10** | **HKCI11** | **HKCI2** | **HKCI4** | **HKCI9** | **HKCIC1** | **HKCIC2** | **HKCIC3** |
| --- | --- | --- | --- | --- | --- | --- | --- | --- |
| **MALAT1** | 9.43 | 13.88 | 36.01 | 19.30 | 24.89 | 17.94 | 13.72 | 16.04 |
| **NEAT1** | 34.80 | 148.88 | 147.13 | 54.08 | 107.85 | 209.73 | 60.97 | 71.68 |
| **PVT1** | 13.11 | 12.34 | 13.87 | 15.15 | 13.12 | 17.78 | 10.64 | 12.16 |

**Supplementary Table 3.** Categories of newly assembled lncRNAs

| **Class code** | **Transcript number** | **Percentage** | **Description** |
| --- | --- | --- | --- |
| j | 227 | 50.44% | At least one splice junction is shared with a reference transcript |
| u | 110 | 24.44% | Unknown, intergenic transcript |
| i | 46 | 10.22% | A transfrag falling entirely within a reference intron |
| x | 42 | 9.33% | Exonic overlap with reference on the opposite strand |
| o | 23 | 5.11% | Generic exonic overlap with a reference transcript |
| c | 2 | 0.44% | Contained by a reference transcript |

**Supplementary Table 4.** Results of differential expression analysis from 81/45 annotated/unannotated tumor-upregulated lncRNAs that have no assembly support from tumor-adjacent samples.

| **Transcript ID** | **DESeq2** | | **EBSeq** | | **edgeR** | | **Mean Transcript Expression  (Expected Counts)** | | **Assembly support** | |
| --- | --- | --- | --- | --- | --- | --- | --- | --- | --- | --- |
|  | **log_2_(fold change)** | **FDR** | **log_2_(fold change)** | **PPDE** | **log_2_(fold change)** | **FDR** | **TCGA-LIHC**  **Tumor** | **TCGA-LIHC**  **Tumor-adjacent** | **TCGA-LIHC**  **Tumor** | **TCGA-LIHC**  **Tumor-adjacent** |
| ENST00000295549.8 | 3.5965 | 0.0000 | 3.5689 | 1.0000 | 3.4659 | 0.0000 | 34.1838 | 2.8670 | 4 | 0 |
| ENST00000354333.5 | 2.5500 | 0.0000 | 2.5371 | 1.0000 | 2.5076 | 0.0000 | 58.1318 | 10.0034 | 3 | 0 |
| ENST00000366109.2 | 1.0969 | 0.0000 | 1.0772 | 0.9997 | 1.0345 | 0.0000 | 16.2891 | 7.7108 | 1 | 0 |
| ENST00000415647.1 | 2.2563 | 0.0000 | 2.2470 | 1.0000 | 2.1932 | 0.0000 | 50.5030 | 10.6273 | 2 | 0 |
| ENST00000417086.5 | 4.5451 | 0.0000 | 4.8219 | 1.0000 | 4.3831 | 0.0000 | 7.6771 | 0.2504 | 6 | 0 |
| ENST00000417262.5 | 1.6415 | 0.0000 | 1.6257 | 0.9997 | 1.5869 | 0.0000 | 57.0092 | 18.4592 | 12 | 0 |
| ENST00000422780.3 | 4.1664 | 0.0000 | 4.2129 | 1.0000 | 4.0481 | 0.0000 | 26.2111 | 1.3928 | 2 | 0 |
| ENST00000426023.1 | 1.6906 | 0.0005 | 3.0113 | 1.0000 | 2.9255 | 0.0000 | 38.2396 | 4.7296 | 3 | 0 |
| ENST00000431558.1 | 1.2442 | 0.0001 | 1.2448 | 0.9998 | 1.2138 | 0.0001 | 42.8822 | 18.0863 | 8 | 0 |
| ENST00000433546.2 | 6.2171 | 0.0000 | 7.7926 | 1.0000 | 6.6312 | 0.0000 | 18.6435 | 0.0694 | 12 | 0 |
| ENST00000438436.2 | 1.0510 | 0.0000 | 1.0517 | 0.9965 | 1.0017 | 0.0004 | 39.9528 | 19.2625 | 9 | 0 |
| ENST00000448869.1 | 3.3591 | 0.0000 | 3.3597 | 1.0000 | 3.2957 | 0.0000 | 83.8171 | 8.1520 | 16 | 0 |
| ENST00000453348.1 | 2.9380 | 0.0000 | 2.9642 | 1.0000 | 2.8457 | 0.0000 | 16.4898 | 2.0972 | 6 | 0 |
| ENST00000455984.1 | 5.9825 | 0.0000 | 6.6016 | 1.0000 | 6.0318 | 0.0000 | 17.9198 | 0.1630 | 14 | 0 |
| ENST00000457147.1 | 1.4639 | 0.0000 | 1.4497 | 0.9997 | 1.3944 | 0.0000 | 12.3235 | 4.5001 | 3 | 0 |
| ENST00000462219.5 | 4.6463 | 0.0057 | 7.8899 | 1.0000 | 5.6069 | 0.0000 | 5.1461 | 0.0000 | 2 | 0 |
| ENST00000464242.1 | 1.1478 | 0.0092 | 1.6519 | 0.9957 | 1.5416 | 0.0006 | 15.6400 | 4.9648 | 3 | 0 |
| ENST00000480284.1 | 4.7726 | 0.0004 | 6.9826 | 1.0000 | 5.9955 | 0.0000 | 17.2501 | 0.1217 | 3 | 0 |
| ENST00000498979.6 | 1.7707 | 0.0000 | 1.7790 | 1.0000 | 1.7397 | 0.0000 | 48.4675 | 14.1102 | 5 | 0 |
| ENST00000502066.6 | 3.8479 | 0.0000 | 3.8793 | 1.0000 | 3.7704 | 0.0000 | 92.7322 | 6.2810 | 19 | 0 |
| ENST00000502390.5 | 1.1538 | 0.0047 | 1.1569 | 0.9925 | 1.1031 | 0.0037 | 333.4956 | 149.5546 | 4 | 0 |
| ENST00000507644.2 | 2.4646 | 0.0004 | 5.8193 | 1.0000 | 5.4528 | 0.0000 | 16.2531 | 0.2733 | 2 | 0 |
| ENST00000507681.1 | 3.6757 | 0.0000 | 3.7076 | 1.0000 | 3.5503 | 0.0000 | 29.1253 | 2.2157 | 5 | 0 |
| ENST00000508572.1 | 4.3116 | 0.0000 | 4.2724 | 1.0000 | 4.0420 | 0.0000 | 14.4671 | 0.7346 | 1 | 0 |
| ENST00000515416.6 | 3.5264 | 0.0000 | 3.4993 | 1.0000 | 3.4767 | 0.0000 | 88.3640 | 7.7978 | 8 | 0 |
| ENST00000515734.2 | 4.0789 | 0.0090 | 8.3602 | 1.0000 | 7.6537 | 0.0000 | 58.6302 | 0.1637 | 6 | 0 |
| ENST00000519762.1 | 6.0379 | 0.0000 | 8.8388 | 1.0000 | 6.7938 | 0.0000 | 15.5008 | 0.0191 | 6 | 0 |
| ENST00000520060.1 | 3.0850 | 0.0000 | 3.0239 | 1.0000 | 2.9628 | 0.0000 | 19.9038 | 2.4312 | 7 | 0 |
| ENST00000520885.1 | 2.4839 | 0.0000 | 2.4950 | 1.0000 | 2.4085 | 0.0000 | 27.0735 | 4.7876 | 8 | 0 |
| ENST00000521028.3 | 6.3557 | 0.0000 | 7.0497 | 1.0000 | 6.3881 | 0.0000 | 27.2416 | 0.1840 | 2 | 0 |
| ENST00000524007.1 | 2.3048 | 0.0000 | 2.2771 | 1.0000 | 2.1929 | 0.0001 | 8.7434 | 1.7866 | 9 | 0 |
| ENST00000528337.1 | 1.5855 | 0.0000 | 1.5760 | 1.0000 | 1.5329 | 0.0000 | 81.3737 | 27.2797 | 6 | 0 |
| ENST00000542314.5 | 2.9831 | 0.0000 | 2.8992 | 1.0000 | 2.8252 | 0.0000 | 16.0463 | 2.1352 | 2 | 0 |
| ENST00000544553.1 | 2.2251 | 0.0000 | 2.2223 | 1.0000 | 2.0893 | 0.0000 | 14.4886 | 3.0878 | 8 | 0 |
| ENST00000550223.5 | 6.0500 | 0.0000 | 6.9583 | 1.0000 | 6.2042 | 0.0000 | 18.8989 | 0.1304 | 10 | 0 |
| ENST00000553330.1 | 4.1216 | 0.0000 | 4.1673 | 1.0000 | 3.8735 | 0.0000 | 8.8262 | 0.4773 | 6 | 0 |
| ENST00000559786.1 | 1.8197 | 0.0001 | 1.8270 | 1.0000 | 1.7336 | 0.0001 | 30.2561 | 8.5173 | 2 | 0 |
| ENST00000561916.2 | 2.0194 | 0.0029 | 1.9969 | 1.0000 | 1.9201 | 0.0012 | 17.3087 | 4.3255 | 1 | 0 |
| ENST00000581579.1 | 2.5156 | 0.0001 | 2.5720 | 1.0000 | 2.4280 | 0.0001 | 16.6768 | 2.7895 | 4 | 0 |
| ENST00000582106.1 | 2.7781 | 0.0000 | 2.7710 | 1.0000 | 2.7121 | 0.0000 | 81.4324 | 11.9150 | 7 | 0 |
| ENST00000582965.1 | 1.9452 | 0.0000 | 1.9625 | 1.0000 | 1.8628 | 0.0000 | 14.9437 | 3.8209 | 2 | 0 |
| ENST00000583962.1 | 1.4018 | 0.0028 | 3.5579 | 1.0000 | 3.5244 | 0.0000 | 36.9934 | 3.1213 | 1 | 0 |
| ENST00000590750.1 | 1.2324 | 0.0009 | 1.1754 | 0.9995 | 1.1134 | 0.0014 | 7.5784 | 3.3472 | 1 | 0 |
| ENST00000591372.1 | 2.2582 | 0.0000 | 2.2632 | 1.0000 | 2.2029 | 0.0000 | 111.4423 | 23.1999 | 9 | 0 |
| ENST00000624162.1 | 1.6406 | 0.0000 | 1.6210 | 1.0000 | 1.5956 | 0.0000 | 32.8050 | 10.6502 | 12 | 0 |
| MHCC.10029.1 | 6.4783 | 0.0000 | 6.3624 | 1.0000 | 6.1924 | 0.0000 | 46.3354 | 0.5485 | 1 | 0 |
| MHCC.10201.2 | 2.5477 | 0.0000 | 2.5062 | 1.0000 | 2.4355 | 0.0000 | 29.7396 | 5.2168 | 4 | 0 |
| MHCC.11884.1 | 2.2647 | 0.0005 | 2.2564 | 1.0000 | 2.2442 | 0.0003 | 62.2608 | 13.0188 | 7 | 0 |
| MHCC.12010.1 | 6.3302 | 0.0000 | 7.8348 | 1.0000 | 6.6169 | 0.0000 | 20.5735 | 0.0754 | 3 | 0 |
| MHCC.12345.1 | 1.0755 | 0.0000 | 1.0691 | 0.9989 | 1.0277 | 0.0000 | 19.8014 | 9.4298 | 1 | 0 |
| MHCC.12346.1 | 4.7016 | 0.0000 | 4.7108 | 1.0000 | 4.5496 | 0.0000 | 26.4900 | 0.9973 | 3 | 0 |
| MHCC.12348.1 | 7.5348 | 0.0000 | 7.4067 | 1.0000 | 7.2803 | 0.0000 | 120.3786 | 0.6947 | 11 | 0 |
| MHCC.12584.2 | 2.7951 | 0.0000 | 2.7903 | 1.0000 | 2.7299 | 0.0000 | 68.6228 | 9.9009 | 4 | 0 |
| MHCC.12640.1 | 6.1248 | 0.0000 | 6.1255 | 1.0000 | 5.9284 | 0.0000 | 45.5914 | 0.6352 | 10 | 0 |
| MHCC.13154.1 | 4.4199 | 0.0000 | 5.1392 | 1.0000 | 4.4654 | 0.0000 | 7.2567 | 0.1915 | 3 | 0 |
| MHCC.13296.1 | 3.3504 | 0.0000 | 5.7017 | 1.0000 | 5.5378 | 0.0000 | 47.7241 | 0.9024 | 2 | 0 |
| MHCC.14603.3 | 1.1004 | 0.0028 | 1.1075 | 0.9981 | 1.0514 | 0.0037 | 61.9566 | 28.7431 | 1 | 0 |
| MHCC.14649.1 | 5.6176 | 0.0000 | 5.8215 | 1.0000 | 5.4958 | 0.0000 | 20.1029 | 0.3410 | 8 | 0 |
| MHCC.1478.2 | 2.5464 | 0.0000 | 2.5237 | 1.0000 | 2.4484 | 0.0000 | 15.8268 | 2.7342 | 2 | 0 |
| MHCC.14970.1 | 1.8731 | 0.0000 | 1.8323 | 1.0000 | 1.7667 | 0.0000 | 16.8598 | 4.7239 | 1 | 0 |
| MHCC.15406.1 | 2.8321 | 0.0000 | 2.8305 | 1.0000 | 2.7558 | 0.0000 | 18.8408 | 2.6300 | 10 | 0 |
| MHCC.15406.2 | 3.3725 | 0.0000 | 3.3864 | 1.0000 | 3.3008 | 0.0000 | 37.6879 | 3.5844 | 7 | 0 |
| MHCC.15641.1 | 4.4392 | 0.0000 | 4.4452 | 1.0000 | 4.3347 | 0.0000 | 42.0364 | 1.9156 | 2 | 0 |
| MHCC.15666.1 | 2.9018 | 0.0000 | 2.9203 | 1.0000 | 2.8473 | 0.0000 | 36.6485 | 4.8286 | 1 | 0 |
| MHCC.15704.1 | 4.3431 | 0.0000 | 4.3244 | 1.0000 | 4.1975 | 0.0000 | 20.2414 | 0.9963 | 11 | 0 |
| MHCC.16116.1 | 2.7068 | 0.0002 | 2.7046 | 1.0000 | 2.5767 | 0.0001 | 17.0388 | 2.6013 | 7 | 0 |
| MHCC.16267.1 | 3.6522 | 0.0000 | 3.6647 | 1.0000 | 3.5938 | 0.0000 | 79.0293 | 6.2179 | 1 | 0 |
| MHCC.16327.1 | 6.4813 | 0.0000 | 6.8273 | 1.0000 | 6.3332 | 0.0000 | 34.0415 | 0.2851 | 3 | 0 |
| MHCC.16355.1 | 1.4394 | 0.0000 | 1.4608 | 1.0000 | 1.3988 | 0.0000 | 439.7200 | 159.7355 | 4 | 0 |
| MHCC.16395.2 | 1.8734 | 0.0000 | 1.8998 | 1.0000 | 1.8095 | 0.0000 | 18.7666 | 5.0132 | 3 | 0 |
| MHCC.16663.5 | 2.1076 | 0.0000 | 2.0735 | 1.0000 | 2.0201 | 0.0000 | 14.8826 | 3.5222 | 1 | 0 |
| MHCC.16891.3 | 3.1038 | 0.0027 | 3.1100 | 1.0000 | 3.0521 | 0.0001 | 49.6000 | 5.7255 | 1 | 0 |
| MHCC.17031.1 | 3.0397 | 0.0001 | 3.0347 | 1.0000 | 2.9568 | 0.0000 | 33.4132 | 4.0645 | 7 | 0 |
| MHCC.17330.1 | 3.0612 | 0.0000 | 4.5767 | 1.0000 | 4.3510 | 0.0000 | 23.6360 | 0.9763 | 6 | 0 |
| MHCC.17345.1 | 1.8527 | 0.0000 | 1.8565 | 1.0000 | 1.8110 | 0.0000 | 30.9880 | 8.5439 | 3 | 0 |
| MHCC.17346.1 | 6.6154 | 0.0000 | 6.5534 | 1.0000 | 6.3311 | 0.0000 | 66.1313 | 0.6895 | 4 | 0 |
| MHCC.17430.2 | 5.4324 | 0.0000 | 6.9807 | 1.0000 | 5.9311 | 0.0000 | 9.6968 | 0.0588 | 5 | 0 |
| MHCC.1745.1 | 5.7223 | 0.0000 | 5.7086 | 1.0000 | 5.5892 | 0.0000 | 93.6417 | 1.7761 | 2 | 0 |
| MHCC.1751.1 | 5.6260 | 0.0000 | 5.5423 | 1.0000 | 5.3978 | 0.0000 | 40.8641 | 0.8624 | 2 | 0 |
| MHCC.17769.1 | 4.4626 | 0.0000 | 4.6322 | 1.0000 | 4.2818 | 0.0000 | 12.9043 | 0.5062 | 6 | 0 |
| MHCC.18007.1 | 3.7326 | 0.0000 | 3.7298 | 1.0000 | 3.5980 | 0.0000 | 24.0288 | 1.7944 | 5 | 0 |
| MHCC.18012.1 | 1.1848 | 0.0003 | 1.1558 | 0.9962 | 1.1515 | 0.0005 | 19.7145 | 8.8385 | 8 | 0 |
| MHCC.18125.1 | 2.6342 | 0.0000 | 3.3045 | 1.0000 | 3.0582 | 0.0000 | 5.7619 | 0.5699 | 1 | 0 |
| MHCC.18676.1 | 5.9568 | 0.0000 | 5.9816 | 1.0000 | 5.8328 | 0.0000 | 112.5366 | 1.7664 | 16 | 0 |
| MHCC.18792.1 | 2.7191 | 0.0001 | 2.7288 | 1.0000 | 2.5959 | 0.0000 | 13.8053 | 2.0672 | 1 | 0 |
| MHCC.18809.1 | 3.1374 | 0.0000 | 3.1057 | 1.0000 | 3.0863 | 0.0000 | 39.3831 | 4.5592 | 8 | 0 |
| MHCC.18911.3 | 3.3959 | 0.0000 | 3.4764 | 1.0000 | 3.1922 | 0.0000 | 6.3773 | 0.5595 | 1 | 0 |
| MHCC.18911.4 | 2.8018 | 0.0005 | 2.6788 | 1.0000 | 2.5297 | 0.0001 | 4.3894 | 0.6702 | 1 | 0 |
| MHCC.19497.1 | 3.3250 | 0.0006 | 4.6800 | 1.0000 | 4.1390 | 0.0000 | 6.0897 | 0.2234 | 1 | 0 |
| MHCC.20001.3 | 2.3428 | 0.0001 | 2.2357 | 1.0000 | 2.1987 | 0.0001 | 4.5927 | 0.9580 | 3 | 0 |
| MHCC.20085.1 | 7.0685 | 0.0000 | 7.0260 | 1.0000 | 6.8833 | 0.0000 | 204.7776 | 1.5533 | 14 | 0 |
| MHCC.20377.1 | 1.6386 | 0.0000 | 2.5537 | 1.0000 | 2.4833 | 0.0000 | 245.5873 | 41.8157 | 1 | 0 |
| MHCC.20392.1 | 2.6995 | 0.0000 | 2.6651 | 1.0000 | 2.6385 | 0.0000 | 17.7210 | 2.7814 | 2 | 0 |
| MHCC.20405.2 | 2.4683 | 0.0002 | 2.4027 | 1.0000 | 2.2912 | 0.0001 | 5.1092 | 0.9485 | 2 | 0 |
| MHCC.2057.1 | 2.9537 | 0.0000 | 2.9583 | 1.0000 | 2.9045 | 0.0000 | 102.8998 | 13.2270 | 1 | 0 |
| MHCC.20685.1 | 3.5848 | 0.0000 | 3.5796 | 1.0000 | 3.4008 | 0.0000 | 17.0098 | 1.4092 | 3 | 0 |
| MHCC.20740.2 | 4.9604 | 0.0000 | 4.9293 | 1.0000 | 4.7587 | 0.0000 | 32.9049 | 1.0624 | 1 | 0 |
| MHCC.20766.3 | 1.7747 | 0.0023 | 2.3147 | 1.0000 | 2.2647 | 0.0001 | 26.2434 | 5.2575 | 6 | 0 |
| MHCC.20768.1 | 2.7676 | 0.0000 | 2.7072 | 1.0000 | 2.6035 | 0.0000 | 10.4515 | 1.5851 | 2 | 0 |
| MHCC.20806.1 | 3.9485 | 0.0000 | 3.8839 | 1.0000 | 3.7592 | 0.0000 | 10.3295 | 0.6859 | 2 | 0 |
| MHCC.20835.1 | 3.4364 | 0.0000 | 3.4342 | 1.0000 | 3.3398 | 0.0000 | 28.5860 | 2.6311 | 3 | 0 |
| MHCC.20970.2 | 4.8459 | 0.0000 | 7.2265 | 1.0000 | 5.5593 | 0.0000 | 6.7622 | 0.0235 | 2 | 0 |
| MHCC.21206.1 | 1.6153 | 0.0074 | 2.5250 | 1.0000 | 2.4508 | 0.0001 | 6.3394 | 1.0834 | 1 | 0 |
| MHCC.2151.1 | 1.2340 | 0.0000 | 1.2411 | 1.0000 | 1.2012 | 0.0000 | 28.2230 | 11.9271 | 1 | 0 |
| MHCC.21867.1 | 1.3806 | 0.0000 | 1.3955 | 1.0000 | 1.3510 | 0.0000 | 39.3764 | 14.9584 | 1 | 0 |
| MHCC.22073.1 | 7.1971 | 0.0000 | 7.3083 | 1.0000 | 6.9908 | 0.0000 | 71.7670 | 0.4381 | 12 | 0 |
| MHCC.22158.1 | 1.5205 | 0.0002 | 1.4955 | 1.0000 | 1.4531 | 0.0004 | 15.2499 | 5.3988 | 1 | 0 |
| MHCC.2268.1 | 3.5460 | 0.0000 | 3.5433 | 1.0000 | 3.5002 | 0.0000 | 42.1306 | 3.5937 | 1 | 0 |
| MHCC.2335.1 | 1.6085 | 0.0000 | 1.5822 | 1.0000 | 1.5521 | 0.0000 | 16.4315 | 5.4779 | 5 | 0 |
| MHCC.2583.1 | 4.5106 | 0.0000 | 4.4819 | 1.0000 | 4.4121 | 0.0000 | 46.8411 | 2.0822 | 6 | 0 |
| MHCC.3312.1 | 1.5191 | 0.0000 | 1.5156 | 1.0000 | 1.4792 | 0.0000 | 70.8861 | 24.7821 | 1 | 0 |
| MHCC.424.1 | 3.8270 | 0.0000 | 3.7986 | 1.0000 | 3.7494 | 0.0000 | 37.6414 | 2.6914 | 8 | 0 |
| MHCC.4295.1 | 1.7652 | 0.0003 | 2.8362 | 1.0000 | 2.7349 | 0.0000 | 12.8298 | 1.7838 | 2 | 0 |
| MHCC.4504.1 | 3.1909 | 0.0002 | 5.6876 | 1.0000 | 5.5438 | 0.0000 | 181.8219 | 3.5133 | 2 | 0 |
| MHCC.5375.5 | 6.2387 | 0.0000 | 6.7996 | 1.0000 | 6.1867 | 0.0000 | 23.9712 | 0.1973 | 9 | 0 |
| MHCC.5528.1 | 5.0316 | 0.0000 | 6.2209 | 1.0000 | 5.9397 | 0.0000 | 42.6616 | 0.5574 | 6 | 0 |
| MHCC.5943.2 | 3.9748 | 0.0000 | 3.9682 | 1.0000 | 3.8941 | 0.0000 | 31.1556 | 1.9702 | 1 | 0 |
| MHCC.6098.1 | 3.6913 | 0.0000 | 3.6556 | 1.0000 | 3.5738 | 0.0000 | 22.3943 | 1.7633 | 1 | 0 |
| MHCC.6297.1 | 2.7305 | 0.0000 | 2.6908 | 1.0000 | 2.5984 | 0.0000 | 11.3531 | 1.7459 | 1 | 0 |
| MHCC.6452.2 | 3.1471 | 0.0000 | 4.2358 | 1.0000 | 3.8564 | 0.0000 | 7.9841 | 0.4098 | 1 | 0 |
| MHCC.6453.1 | 2.1132 | 0.0000 | 2.1005 | 1.0000 | 2.0329 | 0.0000 | 18.5304 | 4.3070 | 1 | 0 |
| MHCC.6573.1 | 5.5383 | 0.0000 | 7.9942 | 1.0000 | 6.2332 | 0.0000 | 9.7497 | 0.0235 | 1 | 0 |
| MHCC.7657.1 | 1.4508 | 0.0000 | 1.3887 | 0.9998 | 1.3859 | 0.0000 | 21.1683 | 8.0755 | 6 | 0 |
| MHCC.8599.1 | 2.0460 | 0.0001 | 2.0647 | 1.0000 | 1.9690 | 0.0002 | 24.6784 | 5.8879 | 10 | 0 |
| MHCC.9567.1 | 5.3900 | 0.0000 | 6.6732 | 1.0000 | 5.7127 | 0.0000 | 10.0870 | 0.0842 | 2 | 0 |
| MHCC.9859.1 | 2.6260 | 0.0000 | 2.5531 | 1.0000 | 2.4591 | 0.0000 | 9.1031 | 1.5388 | 1 | 0 |

**Supplementary Table 5.** List of 28 HCC-associated lncRNA transcripts that correspond to 26 lncRNA genes.

| Gene ID | Gene Symbol | Transcript ID |
| --- | --- | --- |
| ENSG00000203650.8 | LINC01285 | MHCC.22158.1 |
| ENSG00000226328.6 | NUP50-AS1 | MHCC.14603.3 |
| ENSG00000232498.1 | AL136987.1 | MHCC.1751.1 |
| ENSG00000232586.1 | KIAA1614-AS1 | ENST00000415647.1 |
| ENSG00000236751.1 | LINC01186 | MHCC.21867.1 |
| ENSG00000236947.5 | AL139412.1 | MHCC.1478.2 |
| ENSG00000245248.7 | USP2-AS1 | ENST00000498979.6 |
| ENSG00000249413.2 | AC116049.2 | ENST00000508572.1 |
| ENSG00000249464.5 | LINC01091 | MHCC.16663.5 |
| ENSG00000250874.1 | AC010595.1 | MHCC.17345.1 |
| ENSG00000251026.1 | LINC02163 | MHCC.17430.2 |
| ENSG00000251138.6 | AC090502.1 | ENST00000515416.6 |
| ENSG00000253320.5 | AZIN1-AS1 | ENST00000524007.1 |
| ENSG00000253395.1 | AP003469.2 | MHCC.20685.1 |
| ENSG00000253819.1 | LINC01151 | MHCC.20766.3 |
| ENSG00000253877.5 | LINC01608 | MHCC.20740.2 |
| ENSG00000254101.5 | LINC02055 | ENST00000520060.1; MHCC.20835.1 |
| ENSG00000255857.5 | PXN-AS1 | ENST00000542314.5 |
| ENSG00000266402.3 | SNHG25 | ENST00000582965.1 |
| ENSG00000273058.2 | AL359921.2 | MHCC.2151.1 |
| MHCC.12010 | -- | MHCC.12010.1 |
| MHCC.12346 | -- | MHCC.12346.1 |
| MHCC.15406 | -- | MHCC.15406.1; MHCC.15406.2 |
| MHCC.16327 | -- | MHCC.16327.1 |
| MHCC.17346 | -- | MHCC.17346.1 |
| MHCC.3312 | -- | MHCC.3312.1 |

**Supplementary Table 6.** Status of 6 unannotated nuclear-enriched HCC-associated lncRNAs in MiTranscriptome Database

| Gene ID | Genomic coordinates (GRCh38) | Genomic coordinates (GRCh37) | MiTranscriptome Gene ID | MiTranscriptome  Gene function name | Associated  Cancer Type(s) |
| --- | --- | --- | --- | --- | --- |
| MHCC.17346 | chr5:86353759-86570641 (-) | chr5:85649577-85866458 (-) | G066586 | LVCAT3.4 | Liver |
| MHCC.16327 | chr4:65206442-65241885 (-) | chr4:66072160-66107603 (-) | G061385 | LVCAT4.2 | Liver |
| MHCC.12346 | chr2:78456562-78541870 (-) | chr2:78683688-78768996 (-) | G044325 | N/A | N/A |
| MHCC.12010 | chr2:29126294-29130415 (-) | chr2:29349160-29353281 (-) | G042929 | N/A | N/A |
| MHCC.15406 | chr3:115788475-115792525 (-) | chr3:115507322-115511372 (-) | G057145 | N/A | N/A |
| MHCC.3312 | chr11:26589402-26598087 (-) | chr11:26610949-26619634 (-) | G013016 | N/A | N/A |

**Supplementary Table 7.** Mean expression levels (in TPM) of nuclear-enriched HCC-associated lncRNAs in TCGA-LIHC patient tumor samples grouped by their diagnosed etiologies (HBV, HCV and NASH)

| **LncRNA Gene ID** | **HBV etiology**  **(7 samples)** | **HCV etiology**  **(5 samples)** | **NASH etiology**  **(3 samples)** |
| --- | --- | --- | --- |
| ENSG00000203650.8 | 0.9661 | 1.1047 | 0.5960 |
| ENSG00000226328.6 | 10.9525 | 6.0563 | 10.3724 |
| ENSG00000232498.1 | 1.6669 | 2.0478 | 2.1652 |
| ENSG00000232586.1 | 1.3049 | 0.7801 | 0.4884 |
| ENSG00000236751.1 | 1.8129 | 1.6895 | 0.4937 |
| ENSG00000236947.5 | 2.8594 | 0.9829 | 1.6002 |
| ENSG00000245248.7 | 1.0172 | 1.5649 | 1.3856 |
| ENSG00000249413.2 | 0.1960 | 1.5716 | 0.8627 |
| ENSG00000249464.5 | 0.4817 | 1.5940 | 0.2469 |
| ENSG00000250874.1 | 0.6360 | 0.1834 | 0.6018 |
| ENSG00000251026.1 | 1.6007 | 0.3001 | 2.5553 |
| ENSG00000251138.6 | 2.2251 | 0.7925 | 3.9314 |
| ENSG00000253320.5 | 7.6097 | 5.3868 | 5.4279 |
| ENSG00000253395.1 | 1.0221 | 0.3854 | 0.2479 |
| ENSG00000253819.1 | 4.8547 | 2.7822 | 4.3869 |
| ENSG00000253877.5 | 0.1904 | 0.6618 | 0.9281 |
| ENSG00000254101.5 | 2.8375 | 0.9296 | 2.7909 |
| ENSG00000255857.5 | 8.6783 | 6.5234 | 4.6658 |
| ENSG00000266402.3 | 16.6213 | 10.1362 | 6.4204 |
| ENSG00000273058.2 | 1.6762 | 2.5203 | 1.0023 |
| MHCC.12010 | 0.5113 | 0.3876 | 0.2896 |
| MHCC.12346 | 3.1157 | 0.6389 | 1.9275 |
| MHCC.15406 | 3.2797 | 0.7830 | 3.6262 |
| MHCC.16327 | 0.2918 | 0.3010 | 1.4892 |
| MHCC.17346 | 0.6945 | 0.5049 | 1.2825 |
| MHCC.3312 | 0.3130 | 0.2853 | 0.3818 |

**Supplementary Table 8.** Mean expression levels (in TPM) of nuclear-enriched HCC-associated lncRNAs in HKCI cell line samples grouped by their associated etiologies (HBV, HCV and NASH)

| **LncRNA Gene ID** | **HBV etiology**  **(HKCI-4,9,11)** | **HCV etiology**  **(HKCI-C1,C2,C3)** | **NASH etiology**  **(HKCI-2,10)** |
| --- | --- | --- | --- |
| ENSG00000203650.8 | 1.0848 | 0.7754 | 0.3195 |
| ENSG00000226328.6 | 3.0508 | 2.5235 | 2.4053 |
| ENSG00000232498.1 | 2.0662 | 2.0002 | 4.2662 |
| ENSG00000232586.1 | 0.3572 | 0.5025 | 0.4625 |
| ENSG00000236751.1 | 1.9174 | 1.4573 | 1.0985 |
| ENSG00000236947.5 | 1.2848 | 1.1159 | 0.7516 |
| ENSG00000245248.7 | 0.6064 | 0.6759 | 0.4716 |
| ENSG00000249413.2 | 0.4691 | 0.7106 | 0.9102 |
| ENSG00000249464.5 | 3.0966 | 1.6934 | 0.1439 |
| ENSG00000250874.1 | 1.3527 | 0.3751 | 0.6148 |
| ENSG00000251026.1 | 5.6664 | 3.0432 | 2.1229 |
| ENSG00000251138.6 | 8.1349 | 0.6692 | 7.4847 |
| ENSG00000253320.5 | 5.2639 | 3.4979 | 1.7760 |
| ENSG00000253395.1 | 0.4701 | 0.1955 | 0.2348 |
| ENSG00000253819.1 | 2.7560 | 1.2133 | 0.5462 |
| ENSG00000253877.5 | 3.4632 | 4.5044 | 0.9799 |
| ENSG00000254101.5 | 1.0664 | 1.5477 | 1.0038 |
| ENSG00000255857.5 | 2.9859 | 1.7158 | 2.6286 |
| ENSG00000266402.3 | 11.4974 | 16.0058 | 33.2719 |
| ENSG00000273058.2 | 0.4186 | 0.5075 | 0.3663 |
| MHCC.12010 | 0.6415 | 0.0904 | 0.3316 |
| MHCC.12346 | 1.8718 | 0.3856 | 1.9177 |
| MHCC.15406 | 0.4803 | 2.5022 | 0.8809 |
| MHCC.16327 | 0.3060 | 0.7219 | 0.5519 |
| MHCC.17346 | 2.6894 | 0.4030 | 1.7779 |
| MHCC.3312 | 1.0239 | 1.6646 | 1.0790 |

**References**

Hauber, I., Gulle, H., Wolf, H. M., Maris, M., Eggenbauer, H., and Eibl, M. M. (1995). Molecular characterization of major histocompatibility complex class II gene expression and demonstration of antigen-specific T cell response indicate a new phenotype in class II-deficient patients. *J. Exp. Med.* 181, 1411–23. doi:10.1084/jem.181.4.1411.

Meseure, D., Vacher, S., Lallemand, F., Alsibai, K. D., Hatem, R., Chemlali, W., et al. (2016). Prognostic value of a newly identified MALAT1 alternatively spliced transcript in breast cancer. *Br. J. Cancer* 114, 1395–404. doi:10.1038/bjc.2016.123.

Vandesomlele, J. (2002). Accurate normalization of real-time quantitative RT-PCR data. *Genome Biol.* 3, 1–12. doi:10.1186/gb-2002-3-7-research0034.
